# Emergency contraceptive knowledge, attitudes and practices among female students at the University of Botswana: A descriptive survey

**DOI:** 10.4102/phcfm.v10i1.1674

**Published:** 2018-09-06

**Authors:** Bobby Kgosiemang, Julia Blitz

**Affiliations:** 1 Division of Family Medicine and Primary Care, Stellenbosch University, South Africa

## Abstract

**Background:**

Unintended pregnancies are associated with unsafe abortions and maternal deaths, particularly in countries such as Botswana, where abortion is illegal. Many of these unwanted pregnancies could be avoided by using emergency contraception, which is widely available in Botswana.

**Aim:**

To assess the level of knowledge, attitudes and practices of female students with regard to emergency contraception at the University of Botswana.

**Setting:**

Students from University of Botswana, Gaborone, Botswana.

**Methods:**

A descriptive survey among 371 students selected from all eight faculties at the university. Data were collected using a self-administered questionnaire and analysed using the Statistical Package for Social Sciences.

**Results:**

The mean age was 20.6 years (SD 1.62), 58% were sexually active, 22% had used emergency contraception and 52% of pregnancies were unintended. Of the total respondents, 95% replied that they had heard of emergency contraception; however, only 53% were considered to have good knowledge, and 55% had negative attitudes towards its use. Students from urban areas had better knowledge than their rural counterparts (*p* = 0.020). Better knowledge of emergency contraception was associated with more positive attitudes towards actual use (*p* < 0.001). Older students (*p* < 0.001) and those in higher years of study (*p* = 0.001) were more likely to have used emergency contraception.

**Conclusion:**

Although awareness of emergency contraception was high, level of knowledge and intention to use were low. There is a need for a targeted health education programme to provide accurate information about emergency contraception.

## Introduction

Emergency contraception (EC) is a contraceptive method used to prevent pregnancy after a known or suspected failure of contraception or unprotected intercourse, including sexual assault. Emergency contraception hinders or delays ovulation, prevents fertilisation and may affect implantation, but does not disrupt an already established pregnancy.^1^ The two types of contraception that are widely used are oral hormonal tablets and insertion of an intrauterine device (IUD). Hormonal tablets are widely referred to as the ‘morning-after pill’ or ‘second chance’. In Botswana, the commonest EC prescribed is two tablets of levonorgestrel 0.75 mg; however, the IUD method is also available, especially in the private sector. Emergency contraception can reduce the risk of pregnancy after unprotected sexual intercourse or contraceptive failure by between 75% and 99% if taken within 72 h of sexual intercourse.^2^ There is also a favourable success rate when taken between 72 and 120 h after sexual intercourse.^3^

Every year, unplanned pregnancies lead to at least 50 million abortions worldwide and result in approximately 80 000 maternal deaths.^4^ Around 25 million unsafe abortions take place each year, almost all in developing countries, and the risk of dying is highest in Africa.^5^ In countries where abortion is illegal, such as Botswana, women with unintended or unwanted pregnancies tend to seek clandestine and unsafe abortion services. Unsafe abortion has been a major problem in Botswana, and 13% of maternal deaths are attributed to sepsis and 4.3% specifically to septic miscarriage.^6^ The morbidity and mortality report from 2007 to 2011 in Botswana showed that 22% of maternal deaths were attributed to abortion of which 89% were caused by sepsis and 11% from haemorrhage.^7^ This high maternal deaths occur despite all forms of contraception, including EC being free in Botswana. Statistics from the World Bank has shown that in 2008 the contraceptive prevalence rate was low at 52.8%.^8^ Lack of knowledge about EC and how to access it in low-income countries has contributed to maternal morbidity and mortality.^4^

University life for many students represents a move towards independence from parental supervision, new friendships and a chance to experience romantic or sexual relationships. Many sexually active female students in Botswana engage in unprotected sexual intercourse, have multiple partners or intergenerational sexual relationships.^9^ Between 45% and 51% of pregnancies at African universities are unintended and between 22% and 32% of pregnant students induce abortion.^10,11^ Similar studies have not been performed in Botswana and may be difficult because of abortion being illegal.

Although EC is available in Botswana, there has been little research into how widely people are aware of it and their attitudes towards it. The aim of this study was to assess the level of knowledge, attitudes and practice of female students at the University of Botswana towards EC.

## Methods

### Study design

A descriptive survey was conducted using a self-administered questionnaire.

### Setting

The University of Botswana is situated in Gaborone, Botswana. The presumed enrolment figure for the academic year 2015–2016 (University of Botswana, statistics office, personal communication, October and/or November 2015) was approximately 18 000, of which 10 000 were women and 8000 were men. The university offers undergraduate and postgraduate qualifications, degrees and diplomas, with academic teaching continuing during both the day and the evenings. Presumed figures for undergraduate and postgraduate studies were 16 500 and 1500, respectively. There are eight faculties: Business, Education, Engineering and Technology, Graduate Studies, Health Sciences, Humanities, Science and Social Sciences. Students can access EC via the student health services or nearby government clinics.

**FIGURE 1:**
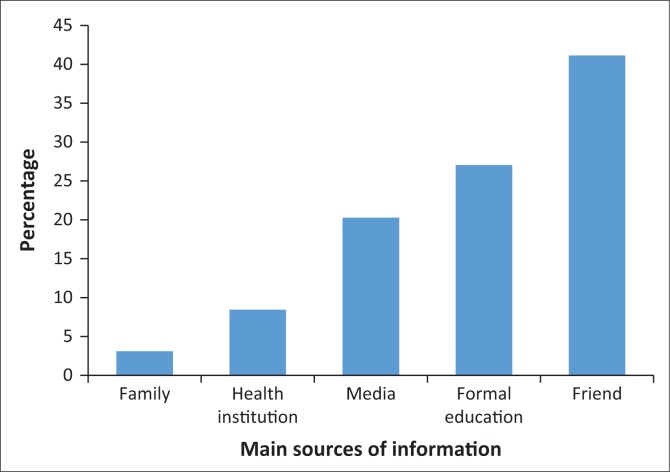
Main sources of information on emergency contraceptive among respondents who have heard about it (*N* = 352).

### Study population and sampling strategy

The study population was full-time female students who attended day classes. Students who attended part-time and distance learners were excluded as they were difficult to access. A sample size of 371 was determined, using an online sample size calculator^12^ and assuming 50% of students would be aware of EC and that this would be measured with a 5% error across 95% confidence intervals. The sample was selected equally from the eight faculties by randomly selecting classes (between 09:00 and 12:00) on specified dates and then inviting all female students to complete the questionnaire until the sample size was obtained.

### Data collection

Data were collected using a self-administered paper-based questionnaire (see supplementary file) between November 2015 and March 2016. The questionnaire had been used previously in Ethiopia and adapted to the local context.^11,13^ Questions were presented in English as this was the language of tuition at the university. The questionnaire asked about socio-demographic and academic characteristics (age, marital status, religion, place of origin, year of study at university), as well as knowledge, attitudes and practice towards EC. The questionnaire was then piloted on 10 students at Botho University in Gaborone, Botswana, which offers both undergraduate and postgraduate courses and is comparable to the University of Botswana in terms of student characteristics (age, place of origin, etc.) but has a smaller student intake. Two research assistants were trained to administer the questionnaire.

### Data analysis

Data were entered into an Excel spreadsheet, prior to analysis in the Statistical Package for Social Sciences version 20.0. Data from incomplete questionnaires were excluded, and, therefore, there may be a slightly different denominator for each variable. For descriptive statistical analysis, results were expressed in terms of frequencies and percentages.

For each respondent, the number of correct answers to the knowledge questions was used to determine the overall percentage of correct answers. Good knowledge was classified as having 50% or more of the answers correct.

For each respondent, the mean attitudinal score was calculated using a three-point scale (0 = no opinion, 1 = disagree and 2 = agree) for each of the three attitudinal statements. A score of 1.5 or above was interpreted as having a ‘positive’ attitude towards EC, and from this, the frequency and percentage of all respondents with a ‘positive’ or ‘negative’ attitude could be determined.

Associations between socio-demographic or academic variables with knowledge, attitudes and practice of EC were initially analysed using the chi-square test.

## Ethical Consideration

Ethical permission for the study was obtained from the Institutional Review Board of the University of Botswana (Ref No: RES/IRB/195), Ministry of Health in Botswana (Ref No: HPDME: 13/18/1 vol. X [38]) and the Health Research Ethics Committee of Stellenbosch University (HREC Reference No: S14/09/186). Ethical permission for the pilot project was obtained from the Research and Consultancy Department of Botho University.

## Results

### Socio-demographic and academic characteristics

A total of 371 female students completed the questionnaire. Table 1 summarises the socio-demographic and academic characteristics of the respondents. Age was normally distributed, and the mean age was 20.6 years (SD 1.62). The majority of the students were single, from rural areas, of Christian background and studying undergraduate degrees.

**TABLE 1:** Socio-demographic and academic characteristics of female university students (*N* = 371).

Variable	*n*	*%*
**Age (*N* = 367)**		
18–19 years	98	26.7
20–21 years	168	45.8
22–23 years	82	22.3
24 years	19	5.2
**Marital status (*N* = 366)**		
Single	350	95.6
Married	3	0.8
Engaged	13	3.6
**Religion (*N* = 343)**		
Orthodox	17	5.0
Islaam and/or Muslim	2	2.0
Catholic	61	17.8
Protestant or Pentecostal	159	46.4
Other	104	30.3
**Year of study (*N* = 368)**		
First year	121	32.9
Second year	86	23.4
Third year	99	26.9
Fourth year	53	14.4
Fifth year	8	2.2
Postgraduate	1	0.3
**Place of origin (*N* = 366)**		
Urban	151	41.5
Rural	213	58.5

### Knowledge of emergency contraception

Overall, 52.8% of respondents had good knowledge of EC. Only 141 (38.2%) respondents correctly identified the recommended time limit for taking the ‘morning-after pills’ after sexual intercourse, 20.3% the number of doses and 17.9% the time interval between doses. Ruptured condoms during intercourse (62.2%), forced sex or rape (50.8%), missed contraceptive pills (7.8%) and failure of contraceptives (20.8%) were chosen as appropriate situations in which to use EC (Table 2). A missed period was correctly pointed out (98.1%) as an inappropriate situation in which to use the ‘morning-after pill’. Only 34% understood that the use of EC was legal in Botswana.

**TABLE 2:** Knowledge regarding emergency contraception among female university students.

Knowledge assessment questions	*n*	*%*
**Situations when EC should be taken†**		
If condom ruptured during intercourse	230	62.2
When there are missed pills	29	7.8
When forced to have sex and/or raped	188	50.8
When there is failure of contraception	77	20.8
When there is a missed period	7	1.9
I do not know	67	18.2
**Where woman can obtain EC**		
Health facility and pharmacy	294	86
Black market	4	1.2
I do not know	44	12.9
**Recommended time limit**		
Within 24 h after sex	117	31.7
Within 72 h after sex	141	38.2
Within 4–6 days after sex	6	1.6
After a missed period	1	0.3
I do not know	104	28.1
**Effectiveness in preventing pregnancy**		
Highly effective	72	19.4
Effective	99	26.7
Less effective	4	1.1
Not effective at all	2	0.5
I do not know	194	52.3
**Recommended number of doses**		
One dose	43	11.6
Two doses	75	20.3
Three doses	11	3.0
I do not know	241	65.1
**Recommended time between doses**		
6 h apart	25	6.7
12 h apart	66	17.8
24 h apart	12	3.2
I do not know	267	72.1
**Knowledge of EC (summary index)**		
Knowledgeable	196	52.8
Not knowledgeable	175	47.2

EC, emergency contraception; h, hours.

† , Some respondents have multiple responses.

### Attitude towards and willingness to use emergency contraception

Overall, 203 (54.7%) respondents had a negative attitude towards EC, and 226 (61.4%) respondents were either worried or unsure as to whether EC might harm the baby if the pregnancy continued. Although 71.2% believed that EC could prevent unwanted pregnancies, only 45.3% were willing to consider using EC (Table 3).

**TABLE 3:** Percentage distribution of female students by attitude towards emergency contraception.

Indicators of attitude	*n*	*%*
**Believe provision of ECs after an episode of unprotected sex can prevent unwanted pregnancy**		
Yes	261	71.7
No	27	7.4
I do not know	76	20.9
**Believe EC may hurt the baby in case it does not work**		
Yes	142	38.6
No	75	20.4
I do not know	151	41.0
**Willingness to use ECs method in the future**		
Yes	168	45.3
No	105	28.3
I do not know	98	26.4

EC, emergency contraception.

### Emergency contraceptive practices and pregnancy-related characteristics among sexually active female university students

A total of 214 (58.3%) respondents reported having been sexually active, 23 (10.7%) had been pregnant and 12 (52.2%) of those pregnancies were unintended. Only 47 (22.0%) students had used EC, while another 36 (16.8%) reported choosing not to use EC when it might have been indicated. Those that used EC were encouraged by either a friend or partner, while those that choose not to use EC were worried about side effects, transgressing their religious beliefs or did not know how to obtain ECs (Table 4).

**TABLE 4:** Emergency contraceptive practices and pregnancy-related characteristics among sexually active female university students.

Characteristics	*n*	*%*
**Ever had sex (*N* = 367)**		
Yes	214	58.3
No	153	41.7
**Ever been pregnant (*N* = 214)**		
Yes	23	10.7
No	191	89.7
**Ever had unwanted pregnancy (*N* = 23)**		
Yes	12	52.2
No	11	47.8
**Ever used ‘morning-after pill’ (*N* = 214)**		
Yes	47	22.2
No	167	78.8
**Who recommended ‘morning-after pill’ (*N* = 45)**		
Friend	18	40.0
Partner	14	31.0
Health professional	8	17.8
I do not remember	2	4.4
Other	3	6.7
**Ever chosen not to use ‘morning-after pill’ (*N* = 214)**		
Yes	36	16.8
No	178	83.2
**Reason not to use ‘morning-after pill’ (*N* = 33)**†		
I did not know where to find	6	18.2
I did not know about ‘morning-after pills’	6	18.2
Partner opposed	4	12.0
Religious reasons	7	21.2
Fear of side effects	8	24.2
Wanted to be pregnant	3	9.0
Other	5	15.2

† , Multiple responses.

### Determinant factors related to knowledge, attitudes or practice

There was no statistical association between the level of knowledge and age, marital status, religion or year of study. However, those from urban areas had better knowledge than their rural counterparts (*p* = 0.020).

There was no association between age, marital status, religion or year of study and attitude towards use of EC. Students from urban areas have significantly more positive attitudes towards EC (*p* = 0.010), and students with good knowledge were also more likely to have positive attitudes (*p* < 0.001).

Older students and those in higher years of study were more likely to have used EC (*p* < 0.001 and *p* = 0.001). There was no association with marital status, religion or place of origin.

## Discussion

Although almost all students had heard of EC, only about half had good knowledge of how to use or access it, and less than half were willing to use it. Students were concerned about the potential side effects, particularly on the foetus; were worried about transgressing religious beliefs; or did not know how to obtain EC. Students from rural areas were likely to be less knowledgeable and to have more negative attitudes towards EC. Students with better knowledge were more likely to have positive attitudes towards EC and to use EC.

Awareness of EC was much higher among Botswana’s students compared to students from Kwazulu-Natal, South Africa (50%)^10^; Kampala, Uganda (45%)^14^; and Makelle Town, Ethiopia (67%).^11^ Levels of awareness were similar to those reported in Mexico (95%).^15^ A large number of students heard about EC from their friends, but very few had heard about it from health institutions. This finding is in line with other studies conducted in South Africa.^10,16^ In light of this finding, peer education approaches may be useful in increasing EC awareness. It is also clear from this finding that there may be a need for health care providers to provide more information on EC, which could be done routinely as part of reproductive health counselling.

Although awareness of EC was high in this report, accurate knowledge of EC was lacking, which is also consistent with findings from Ethiopia.^11^ This poor knowledge of EC despite very high awareness may be because of the fact that many students’ sourced information from peers. The lack of knowledge of EC use and side effects, concerns associated with cultural and societal beliefs, and misconceptions about its utilisation could be the reasons for the low usage.^17^ Women who live in urban areas may have more opportunities to obtain reproductive health information, and this may explain their better levels of knowledge. A link between better knowledge and more positive attitudes towards using EC was also found in Ethiopia.^11^ It is particularly worrying that 62% of the sample were unclear about the legality of using EC and may have conflated the use of EC with illegal abortion, or have religious concerns about it. Overall, most of the female students had a negative attitude (55%) towards the utilisation of EC as was also found in Ethiopia.^11^

Use of EC was comparable to South African studies which showed utilisation among sexually active female students between 21%^10^ and 28%.^17^ The level of utilisation in these two studies^10,17^ may be influenced by the fact that EC can be obtained easily over the counter in pharmacies by students, while in Botswana one needs a prescription. However, these levels of EC utilisation are higher than 7% in Ethiopia^18^ and 6% in Nigeria.^19^

### Limitations of the study

The data were collected from only one university, and therefore, results may not be generalisable to other universities. Students who have done very well at the Botswana General Certificate for Secondary Education (BGCSE) examination prefer to enrol at the University of Botswana rather than other universities and colleges around the country. Therefore, because these students are ‘smarter’, they are expected to have a better knowledge about scientific issues, including emergency contraception, than those who enroll elsewhere. Furthermore, this result has limited power to be generalised to all youth in the country as only a small proportion have the chance of higher education. Because of the sensitive nature of the study, it is possible that respondents under-reported sexual activity and the use of EC. Postgraduate students are under-represented and, therefore, conclusions about the study may apply only to undergraduate students. Furthermore, because the number of students per faculty is not equal, a multistage sampling technique should have been used.

### Recommendations

Initiatives are needed to improve the knowledge of university students regarding how to use and access EC, as well as to address common misconceptions and beliefs. Health services should take a stronger lead in advocating such information campaigns and contributing to them, as well as ensuring that students can easily access EC at hospitals, pharmacies and student clinics.

Future qualitative research may help to explore attitudes and beliefs and to understand in more depth the barriers to accessing and using EC. However, these recommendations are tentative because as stated above findings are not generalisable.

## Conclusion

Although overall awareness of EC was very high among female students at the University of Botswana, only half had good knowledge of EC and less than half had a positive attitude towards using EC. Use of EC was low and half of those who were pregnant reported that the pregnancy was unintended. Students were worried about EC side effects, transgressing religious beliefs by using EC or did not know how to access EC. Students from urban areas were more likely to have good knowledge of EC, and those with good knowledge of EC and those from urban areas were more likely to have positive attitudes towards using EC. Older students and those from higher academic years of study were more likely to use EC. Accurate knowledge about EC should be made more widely available to students of the University of Botswana, and student health services should take the lead in enabling this and making EC more easily available.
